# A new impetus for guideline development and implementation: construction and evaluation of a toolbox

**DOI:** 10.1186/1748-5908-9-34

**Published:** 2014-03-19

**Authors:** Mirrian AHW Hilbink, Marielle MTJ Ouwens, Jako S Burgers, Rudolf B Kool

**Affiliations:** 1 Scientific Institute for Quality of Healthcare, Radboud university medical center, PO box 9101; Code: 114 IQ healthcare, 6500, HB Nijmegen, The Netherlands; 2 The Dutch College of General Practitioners, Utrecht, The Netherlands

**Keywords:** Guideline development, Guideline implementation, Tools, Toolbox

## Abstract

**Background:**

In the last decade, guideline organizations faced a number of problems, including a lack of standardization in guideline development methods and suboptimal guideline implementation. To contribute to the solution of these problems, we produced a toolbox for guideline development, implementation, revision, and evaluation.

**Methods:**

All relevant guideline organizations in the Netherlands were approached to prioritize the topics. We sent out a questionnaire and discussed the results at an invitational conference. Based on consensus, twelve topics were selected for the development of new tools. Subsequently, working groups were composed for the development of the tools. After development of the tools, their draft versions were pilot tested in 40 guideline projects. Based on the results of the pilot tests, the tools were refined and their final versions were presented.

**Results:**

The vast majority of organizations involved in pilot testing of the tools reported satisfaction with using the tools. Guideline experts involved in pilot testing of the tools proposed a variety of suggestions for the implementation of the tools. The tools are available in Dutch and in English at a web-based platform on guideline development and implementation (http://www.ha-ring.nl).

**Conclusions:**

A collaborative approach was used for the development and evaluation of a toolbox for development, implementation, revision, and evaluation of guidelines. This approach yielded a potentially powerful toolbox for improving the quality and implementation of Dutch clinical guidelines. Collaboration between guideline organizations within this project led to stronger linkages, which is useful for enhancing coordination of guideline development and implementation and preventing duplication of efforts. Use of the toolbox could improve quality standards in the Netherlands, and might facilitate the development of high-quality guidelines in other countries as well.

## Background

Since the 1980s, Dutch healthcare professionals have invested much time and resources on development and implementation of hundreds of guidelines [1]. In the 1990s, the Dutch Institute for Healthcare Improvement CBO and the Dutch Cochrane Center took a next step aiming to improve the quality of guideline development by starting a national collaboration among guideline developers. This resulted in a platform involving almost 30 organizations [2]. Currently, almost all those professional organizations are actively developing guidelines. This is encouraged by governmental legislation and regulation, aiming to increase transparency in healthcare quality and costs.

In the last decade, guideline organizations faced new problems [1]. First, both guideline developers and implementation experts reported a lack of methodological support in producing high-quality guidelines and felt a need for specific tools to help them in improving the quality of the guidelines [3]. A second problem is that the development and updating of high-quality guidelines require substantial resources, while government is cutting back spending on guideline development. Therefore, most organizations are under pressure to produce more guidelines in a shorter time with the same quality. Addressing the views and perspectives of patients and the public, for instance, is mandatory but often complex and time-consuming [4]. Third, national guideline developers do not always feel responsible for implementation and may leave it to regional or local groups. Effective implementation, however, often requires a multifaceted national approach as wider national initiatives generally have a more powerful influence than local implementation efforts [5]. However, such a national approach is not always feasible: in locally heterogeneous conditions, for example, implementation activities must be tailored to local barriers and enablers. In the Netherlands, the implementation of guidelines lacks a well-developed infrastructure and sustainable programs [6].

In order to face these problems, the Dutch Government established a Quality Institute in 2012 as an independent body with the aim to improve the quality of Dutch healthcare. The Dutch Quality Institute is convinced that patients, healthcare professionals, and healthcare insurers are experts in defining high-quality healthcare, and that they themselves have to make agreements on quality standards. Those quality standards are central in supporting the government’s vision for a health and social care system focused on delivering the best possible outcomes for patients and civilians. The role and position of the Dutch Quality Institute is comparable to the National Institute for Health and Care Excellence (NICE) in the provision of guidance and advice to improve health and social care in the UK.

In order to fulfill these expectations, quality criteria were formulated for initiating, developing, and implementing guidelines in the Netherlands [7]. In addition, tools could be helpful to meet these quality standards. The objective of this paper is to describe the process of the development and evaluation of a toolbox for development, implementation, revision, and evaluation of guidelines in order to improve quality standards in the Netherlands and to be helpful for other countries.

## Methods

### Topic selection

Based on an analysis of barriers to the development and implementation of guidelines [8], a long list of potential topics for tools was composed. For each potential topic, we searched the literature for existing supportive materials. To prioritize the most relevant topics, we conducted a web-based survey among national guideline developing organizations and opinion leaders in the field of guideline development and implementation (Table 1). One hundred and forty-three online questionnaires were sent out and 74 questionnaires were returned, yielding a response rate of 52%. Responders were fairly equally divided between the approached professional groups. For each topic, the questionnaire included questions on awareness of existing guidance or tools; use of these materials in daily practice; and the extent to which there was a need for new supportive materials or tools. A nine-point response scale was used with answers ranging from ‘strong need’ to ‘no need.’ They were also asked to provide a top-three ranking of most relevant topics for the development of new tools. Finally, participants were asked to suggest additional topics that were not included in the overview of potential topics. In September 2010, we organized a national invitational conference to reach consensus on the topics for new tools. At this conference (attended by 49 responders to the online questionnaire), the results of the survey were presented. Problems as well as possible solutions on topics that scored a strong need for new tools were subsequently discussed in small groups. Finally, consensus was reached about a top ten of topics, added with two topics preselected by the funding organization of our project (cost-effectiveness in guidelines, shared decision making in guidelines). Table 2 provides an overview of the topics that were selected for the development of new tools. Table 2 shows that the selected topics cover the entire guideline development project: three topics specifically relate to the preparation phase of guideline development, three topics pertain to the development phase, and four topics refer to the completion phase. Two topics cover the entire guideline development process, *i.e.*, organization and cooperation in multidisciplinary guideline development and project management in guideline development.

**Table 1:** Organizations involved in the topic selection, tools development and/or pilot testing of the tools

	**Topic selection**	**Tools development**	**Pilot testing**
**Professional society**			
Dutch Order of Medical Specialists (OMS)	+	+	+
Dutch College of General Practitioners (NHG)	+	+	+
Royal Dutch Society for Physical Therapy (KNGF)	+	+	+
Dutch Association of Nurses and Care Providers (V&VN)	+	+	-
Dutch Association of Youth Health Care Doctors (AJN)	+	+	-
Dutch Urological Association (NVU)	-	-	+
**Research institute**			
Netherlands Organisation for Applied Scientific Research (TNO)	+	+	+
Netherlands institute for health services research (NIVEL)	+	+	-
institute for Medical Technology Assessment (iMTA)	+	+	-
**Quality improvement organization**			
Dutch Institute for Healthcare Improvement (CBO)	+	+	+
Dutch Council for Quality of Healthcare	+	+	-
**Knowledge institute**			
Trimbos Institute	+	+	+
Dutch Youth Health Centre (NCJ)	+	+	+
Netherlands Youth Institute (NJi)	+	+	+
**Medical Centre**			
Academic Medical Center (AMC)	+	+	-
Onze Lieve Vrouwe Gasthuis (OLVG)	+	+	-
Radboud University Nijmegen Medical Centre (RUNMC)	+	+	+
University Medical Center Groningen (UMCG)	+	+	-
Leiden University Medical Center (LUMC)	+	+	+
University Medical Center Utrecht	+	+	+
**Educational organizations**			
Maastricht University	+	+	+
Utrecht University	+	+	+
**Disease specific organization**			
Comprehensive Cancer Centre The Netherlands (IKNL)	+	+	+
Dutch Lung Alliance (LAN)	-	-	+
**Other**			
PROVA	+	+	+
Federation of Patients and Consumers Organisations in the Netherlands (NPCF)	+	+	-
Dutch Burns Foundation	-	-	+

**Table 2:** Topics selected for the development of new tools

**Topic**	Phase*
1. Analysis of barriers in clinical care	P
2. Cost-effectiveness in guidelines	P
3. Prevention of improper influence due to conflicts of interest	P
4. International collaboration in guideline development	D
5. Developing population and sex specific recommendations	D
6. Shared decision making in guidelines	D
7. Knowledge gaps in guidelines	C
8. Implementation of guidelines	C
9. Monitoring of guidelines	C
10. Electronic disclosure of guidelines	C
11. Organization and cooperation in multidisciplinary guideline development	P, D, C
12. Project management in guideline development.	P, D, C

*Phase of the guideline development process to which the topic pertains. P: preparation phase; D: development phase; C: completion phase.

### Development of tools

In October 2010, all Dutch organizations involved in guideline development, revision, implementation, and monitoring (n = 24) were asked to delegate professionals with specific expertise on one or more selected topics for tools. Thirteen working groups, each consisting of four or five people from different organizations (Table 1), were composed for the development of the tools. Each working group appointed a chairman, met four to six times face-to-face, and arranged some additional contacts by e-mail, telephone, or video-conferencing. After six months, each group delivered a draft version of the tool. This draft tool was sent to all organizations involved and a large group of experts for comment and review. One hundred and fifty experts submitted valuable suggestions for improvement. The received comments were analyzed and integrated in a next version of the tool.

### Pilot testing of tools

National organizations involved in the development and implementation of clinical practice guidelines were approached to test the tools. Considering their current and future guideline projects, representatives of those organizations selected specific guideline projects for pilot testing of the tools. In 40 guideline projects selected, one or more tools were tested. In total, 18 organizations were involved in the pilot testing of the tools (Table 1). At the start of the evaluation, the project leader and chairman of the guideline group received detailed information on both the content of the tools and the aims of the pilot test. Halfway through the evaluation process, the project leader of the guideline project was contacted by telephone to inquire about the status of the evaluation of the tools. At the end of the evaluation process of the tools, a one-hour face-to-face meeting with the project leader, chairman and one or two other members of the guideline group was arranged. During this meeting, the following topics were discussed: experiences with the use of the tool and intentions to use the tools in future guideline projects; suggestions for improvement of the tool; and suggestions for dissemination and implementation of the tool. Based on the information yielded in these meetings, the tools were revised and their final versions were produced. With the exception of the tool on the prevention of improper influence due to conflicts of interest, the pilot testings of the tools were qualitative evaluations. In order to evaluate the conflicts of interests tool, individual members of guideline projects were approached with the request to complete a web-based questionnaire on their experiences and satisfaction with completing the ‘Declaration of Interests’ form. The participants answered the questions by marking their responses on a five-point Likert Scale.

## Results

### Experiences with the use of the tools and intention to use the tools in future guideline projects

The vast majority of experts involved in pilot testing of the tools reported satisfactory experiences with the use of the tools. The tools provided them the necessary support to deal with particular aspects of the guideline development, revision, implementation, and monitoring process in an improved manner. In particular, tools with a more practical structure, such as checklists and step-by-step plans, received positive feedback during the pilot test. Two tools covering the entire guideline process, *i.e.*, the tools on process management and on project management, were perceived as too broad in scope. Several experts experienced the working methods as described in the tools as more time-consuming compared to their routine procedures. Furthermore, some tools contain rather innovative approaches that differ significantly from common methods of guideline development. For example, the tool for the analysis of barriers in clinical care regards the stages of identifying, analyzing, and ultimately selecting barriers in clinical care as an independent process that occurs before any planned guideline project. Until now, however, the analysis of barriers in clinical care has been part of the guideline development process.

The intention to use the tools in future guideline projects varied among the participating experts and depended on a number of factors, such as the availability and satisfaction with current procedures, the experience of key figures in the guideline development group and the time needed to apply the tool. Most experts involved in pilot testing of the tools had the intention to use one or more tools in future guideline projects, or at least specific parts of the tools that appeared to be most helpful for them.

### Suggestions for improvement of the tools

After pilot testing of the tools, all tools underwent revisions on their content. Some tools were only slightly adapted to clarify their content, whereas a few tools were revised thoroughly based on the experiences during the pilot test. The scope of the tools on process management and project management was narrowed: the content of the tools was restricted to guideline projects, and general information on process and project management was removed. The tool on implementation of guidelines was aligned with the model for effective implementation of Grol *et al.*[9].

### Suggestions for dissemination and implementation of the tools

Experts involved in pilot testing of the tools proposed the following suggestions for dissemination and implementation of the tools.

1. Design of a digital knowledge platform on guideline development and implementation for dissemination of the tools;

2. Publication of the tools in national and international scientific journals;

3. Inclusion of the tools in educational materials and educational meetings for guideline developers and implementation experts;

4. Inclusion of use of the tools in funding requirements for grants on guideline development and implementation;

5. Availability of sufficient resources and financial support to develop, revise, implement, and monitor guidelines according to the approach suggested in the tools.

### Evaluation of the tool on the prevention of improper influence due to conflicts of interest

The questionnaire was sent to 112 members of eight different guideline projects. From the approached members, 50 people (45%) filled in a questionnaire. Ninety percent of the respondents answered that the various relations and interests mentioned in the ‘Declaration of Interests’ form were completely clear for them. Nearly all respondents (94%) considered the form as an exhaustive and comprehensive document that would not miss any relations or interests. The respondents had differing points of view on the contribution of the ‘Declaration of Interests’ form to the actual prevention of improper influence. About one-half of the respondents (56%) answered that the form will enlarge both the awareness and the transparency on improper influence due to conflicts of interests, but considered this as insufficient for the effective prevention of it. Finally, ten respondents (9%) answered that the extent to which improper influence due to conflicts of interests occurs is not measurable with this form. Nevertheless, the large majority of respondents (85%) considered completion of the form as a first important step towards transparency and clarity on possible relations and interests and a useful document for assessment of the risk for improper influence due to conflicts of interest.

### The final toolbox

Twelve tools on guideline development, implementation, revision, and monitoring were produced, according to the following format: title of the tool; background; objective of the tool; content of the tool; and relevant literature. The tools differ in terms of structure: some tools include a step-by-step plan or a checklist, whereas other tools have a more descriptive character. Table 3 summarizes the main characteristics of the tools, *i.e.*, the title of the tool, the objective(s) of the tool, and the structure of the tool. The full-text versions of the tools are available in Dutch and in English via a comprehensive website on guideline development and implementation (http://www.ha-ring.nl).

**Table 3:** Main characteristics of the developed tools

**Title of the tool**	**Objective(s) of the tool**	**Structure of the tool**
Analysis of barriers in clinical care (http://www.ha-ring.nl/en/tool-1)	To support the processes of 1) defining the scope of the guideline; 2) identifying barriers in clinical care; 3) analyzing barriers in clinical care; and 4) selecting and prioritizing barriers in clinical care	Six-step plan for determining, selecting, and addressing barriers in clinical care
Integration of cost-effectiveness in guidelines (http://www.ha-ring.nl/en/tool-2)	1) To demonstrate how cost-effectiveness and/or budget impact can be involved in drafting a guideline	Descriptive text, including taxonomy, general information and recommendations
	2) To offer support in evaluating whether additional analyses are needed and what type of expertise this will require	
Prevention of improper influence due to conflicts of interest (http://www.ha-ring.nl/en/tool-4)	To enlarge transparency about relationships and interests of all proposed stakeholders involved in preparing scientific advisory reports and medical guidelines	‘Declaration of Interests’ form
International collaboration in guideline development (http://www.ha-ring.nl/en/tool-5)	To formulate both conditions for successful international collaboration and steps that guideline developers need to follow when collaborating at an international level	Six-step plan on how to collaborate internationally in developing guidelines
Developing population-specific recommendations (http://www.ha-ring.nl/en/tool-7)	To facilitate the development of recommendations that are as specific as possible	Checklist, containing a number of questions that a guideline development group can ask when formulating recommendations
Integration of shared decision making in guidelines (http://www.ha-ring.nl/en/tool-8)	To promote shared decision making between health care providers and patients when implementing preference-sensitive guideline recommendations in practice	Descriptive text using hyperlinks to background materials and papers
Definition and identification of knowledge gaps in guidelines (http://www.ha-ring.nl/en/tool-9)	1) To facilitate both the identification and definition of knowledge gaps during the guideline development process	Descriptive text, including a definition of knowledge gaps, information on forms of knowledge gaps and criteria for knowledge gaps
	2) To support researchers and grant providers in selecting and funding themes that have a clear relevance to patient care	
Implementation of guidelines (http://www.ha-ring.nl/en/tool-10)	To stress the importance of prompt attention for implementation of the guideline, with the ultimate aim of improving the implementability of the guideline	Checklist, containing a number of points that a guideline development group can consider, which will facilitate the implementation of the guideline recommendations in practice
Monitoring of guidelines (http://www.ha-ring.nl/en/tool-11)	To provide a useful framework for the development of a monitoring plan	Eight-step plan for the construction of a monitoring plan
Electronic disclosure of guidelines (http://www.ha-ring.nl/en/tool-12)	To improve the ability to search and find guidelines	Overview of available Dutch guideline databases; checklist of criteria for improving the ability to search and find guidelines
Organization and cooperation in multidisciplinary guideline development (http://www.ha-ring.nl/en/tool-3)	To provide practical tips for, and examples of, process management, to promote (interprofessional) cooperation in guideline development groups	Descriptive text, including general information, recommendations and examples
Project management in guideline development (http://www.ha-ring.nl/en/tool-6)	To provide tips, instruments and helpful tools for efficient project management when developing or revising guidelines and/or products derived from them	Descriptive text, including tips, tricks and helpful tools for efficient project management

## Discussion

Our project showed that national collaboration among Dutch guideline organizations resulted in a toolbox covering a set of relevant topics on guideline methodology. Experts involved in pilot testing of the tools reported satisfactory experiences with the use of the tools. A recent comparative review of clinical practice guideline development handbooks showed that specific support for evidence based development of guidelines was lacking [3]. This toolbox can have added value to existing supportive materials on guideline development and implementation [10-12], as it elaborates on various aspects of the guideline development process for which there appeared to be a great need for new supportive materials. Because the toolbox covers the complete guideline development project (preparation phase, development phase, and completion phase), it could have a major impact on many aspects of the guideline development process in The Netherlands and in other countries as well.

The tools are available via a comprehensive website on guideline development and implementation (http://www.ha-ring.nl). This digital knowledge platform could be a powerful instrument for improving Dutch clinical guidelines. The recently established Dutch Quality Institute will decide about proposed additions to the knowledge platform and will keep the platform up-to-date. Furthermore, the Dutch Quality Institute is currently developing a national registry and database of clinical practice guidelines using the National Clearinghouse Guidelines and NHS Evidence as examples. The Institute has recommended the use of our tools in order to fulfill the quality criteria for inclusion in the database. This toolbox facilitates the Dutch Quality Institute in improving the quality of healthcare and in providing guidance and advice to improve healthcare as NICE does in the UK.

Several limitations of our work need to be mentioned. First, all tools are based on experts’ opinion on best practices of producing and implementing guidelines. Although everyone involved in the development and pilot testing of the tools are leading experts on the concerning topics, certain comments of other experts contained suggestions for a somewhat different approach. Second, it might be difficult to encourage persons who are highly experienced in the field of guideline development and implementation to use these tools, because they possibly prefer to stick to their own methods and routines. Hopefully, their less experienced colleagues, to whom the tools will be disseminated via educational materials and meetings, will succeed in convincing them of the value of these tools. Finally, the tools on implementation might have limited international generalizability, because countries that are larger and have a more heterogeneous legislation than the Netherlands probably benefit more from an implementation approach tailored to the local context.

Beyond the development of the toolbox, our study also led to stronger linkages between Dutch organizations involved in guideline development and implementation. As a consequence, they established the Collaborative Dutch Association for Excellent Guidelines, called GENEVER, in 2012. The establishment of GENEVER, a paraphrase to a well-known Dutch liquor, has resulted in an intensive exchange of knowledge and information among its members, similar as in the Guidelines International Network (http://www.g-i-n.net). All members of GENEVER are familiar with the tools and willing to support the dissemination of the toolbox and its implementation within the national and international context. Implementation of the tools will facilitate the development of high-quality guidelines, which could result in higher guideline adherence and, ultimately, in improved quality of care.

## Conclusions

A collaborative approach was used for the development and evaluation of a toolbox for development, implementation, revision, and evaluation of guidelines. This approach yielded a potentially powerful toolbox for improving the quality and implementation of Dutch clinical guidelines. Collaboration between guideline organizations within this project led to stronger linkages, which is useful for enhancing coordination of guideline development and implementation and preventing duplication of efforts. Use of the toolbox could improve quality standards in the Netherlands, and might facilitate the development of high-quality guidelines in other countries as well.

### Ethical approval

Ethical approval was not needed for this study.

## Competing interests

All authors declare to have no competing interests.

## Authors’ contributions

JSB was involved in the conception and design of the study and obtained funding. AHWH, MMTJO, JSB and RBK were involved with the acquisition of study data. All authors were responsible for the analysis and interpretation of data. AHWH drafted the manuscript, with critical revision from MMTJO, JSB and RBK. All authors approved the final version of the manuscript.
